# Affinity-matured variants derived from nimotuzumab keep the original fine specificity and exhibit superior biological activity

**DOI:** 10.1038/s41598-019-57279-w

**Published:** 2020-01-27

**Authors:** Yaima Tundidor, Luis F. Ponce, Lisset Chao, Joaquín Solozábal, Michael Hust, Stefan Dübel, Gertrudis Rojas

**Affiliations:** 1 Center of Molecular Immunology, calle 216 esq 15, PO Box 16040, Atabey, Playa, CP, 11300 La Habana, Cuba; 2 Technische Universität Braunschweig, Institute of Biochemistry, Biotechnology and Bioinformatics, Department of Biotechnology, Spielmannstraße 7, 38106 Braunschweig, Germany

**Keywords:** Biochemistry, Biological techniques, Biotechnology, Cancer, Computational biology and bioinformatics, Immunology, Molecular biology

## Abstract

Nimotuzumab is a humanized monoclonal antibody against the Epidermal Growth Factor Receptor with a long history of therapeutic use, recognizing an epitope different from the ones targeted by other antibodies against the same antigen. It is also distinguished by much less toxicity resulting in a better safety profile, which has been attributed to its lower affinity compared to these other antibodies. Nevertheless, the ideal affinity window for optimizing the balance between anti-tumor activity and toxic effects has not been determined. In the current work, the paratope of the phage-displayed nimotuzumab Fab fragment was evolved *in vitro* to obtain affinity-matured variants. Soft-randomization of heavy chain variable region CDRs and phage selection resulted in mutated variants with improved binding ability. Two recombinant antibodies were constructed using these variable regions, which kept the original fine epitope specificity and showed moderate affinity increases against the target (3-4-fold). Such differences were translated into a greatly enhanced inhibitory capacity upon ligand-induced receptor phosphorylation on tumor cells. The new antibodies, named K4 and K5, are valuable tools to explore the role of affinity in nimotuzumab biological properties, and could be used for applications requiring a fine-tuning of the balance between binding to tumor cells and healthy tissues.

## Introduction

The epidermal growth factor receptor (EGF-R) remains one of the best-established targets for anti-tumor therapies. This receptor is involved in cellular processes that contribute to the survival of epithelial cells. Deregulation of the EGF/EGF-R pathway by receptor overexpression or constitutive activation promotes tumor cell proliferation, invasion, and is associated with poor prognosis in cancer^1^. Up to now three anti-EGF-R monoclonal antibodies (mAbs) have been approved for clinical use by the FDA: cetuximab (2004), panitumumab (2006) and necitumumab (2015)^2^. Nimotuzumab recognizes the same target and has a long history of therapeutic use^3^, starting with clinical trials since 1998, and first registered by the Cuban regulatory authorities in 2002^4^. Nimotuzumab is currently approved in Cuba for the treatment of childhood and adult glioma, advanced esophageal cancer, and squamous cell carcinoma of the head and neck, in combination with chemo-radiotherapy or radiotherapy alone, and is also registered in 28 additional countries.

The availability of several mAbs targeting the same tumor antigen can result in different clinical outcomes in terms of therapeutic efficacy, safety, anti-tumor mechanisms, immunogenicity, patients' sub-population that can receive a benefit, and development of resistance to therapy. The major molecular determinants behind such complex landscape of clinical effects are the origin of constant domains (species and isotype), the strength of binding to the target (affinity) and the topology of interaction with the specific antigen region that is recognized (epitope specificity). Properties associated to the nature of constant domains can be readily engineered, because the modular architecture of antibodies allows exchanging and modifying these regions without affecting antigen-targeting ability^5^. On the other hand, affinity and epitope fine specificity both reside in the unique antigen-binding site of each antibody (paratope) and modulating them requires a careful manipulation to avoid losing the valuable properties of the antibody.

In the case of anti-EGF-R mAbs, there are distinctive properties distinguishing their clinical effects. A recent meta-analysis of 65 randomized controlled trials (including 25 994 cancer patients treated with cetuximab, panitumumab or nimotuzumab) indicated that the use of anti-EGF-R mAbs significantly increases the risk of developing skin toxicity (rash, hand–foot syndrome, dry skin and oral mucositis), but patients receiving nimotuzumab have the lowest risk among all^6^. In fact, no signs of severe toxicity have been observed upon nimotuzumab administration neither in pre-clinical studies in monkeys^7^ nor in clinical trials^8–11^. The absence of serious adverse events has allowed the continued use of nimotuzumab for long time periods (several months and even years) in multiple patients^12,13^. Nimotuzumab is thus the only anti-EGF-R mAb that can be chronically used^3^, in contrast to cetuximab and panitumumab, for which skin toxicity often determines the need of dose reduction or therapy discontinuation. Such a favorable safety profile has been attributed to its lower affinity (K_D_ in the order of 10^−8^ mol/L), that results in maximum uptake by EGF-R-overexpressing tumors and negligible binding to normal epithelial cells displaying basal EGF-R levels^8^. It has been indeed demonstrated that nimotuzumab, unlike higher intrinsic affinity mAbs such as cetuximab, is strongly dependent on avidity effects. Bivalent binding of both Fab arms to two target molecules on the same cell is essential for its function, and this can only be achieved in conditions of high EGF-R cell surface density, providing a molecular explanation for its selective activity on tumor versus normal cells^14^.

However, the influence of affinity to EGF-R on global clinical outcome is not so simple. Although higher affinity is linked to high skin toxicity, multiple studies show a direct correlation between the severity of skin rash and the greatest benefit in patients’ survival^15^. A careful review of clinical studies shows that nimotuzumab benefit seems to be biased towards the subset of patients with higher EGF-R levels^3^. Such a selectivity can be similarly attributed to the lower affinity of nimotuzumab compared to other anti-EGF-R mAbs. The ideal affinity window resulting in an optimal ratio between effective tumor targeting and minimal effects on normal epithelial cells is thus not known, and could be explored through *in vitro* evolution of variants of the same antibody with different affinities.

Fine epitope specificity also distinguishes the different anti-EGF-R. While several of them recognize partially overlapping areas on EGF-R domain III (one of the domains responsible of ligand binding), the key residues (those making the largest energetic contribution to the interactions with each antibody) are clearly different^16,17^. The functional relevance of these subtle differences has been highlighted by the discovery of an emerging mutation in the extracellular domain of EGF-R on tumor cells upon cetuximab treatment, both *in vitro* and *in vivo*, that determines loss of cetuximab binding and subsequent resistance to therapy. This mechanism of tumor escape is linked to epitope loss rather than to target antigen loss, and is specific for a given therapeutic agent, while other antibodies against the same target can be still effective^18^. This initial finding was expanded by the description of additional mutations in the EGF-R extracellular domain that arise upon treatment and mediate resistance to cetuximab and panitumumab^19–21^. Remarkably, some of these mutations affecting cetuximab binding (I491M/I467M, S464L/S440L, S492R/S468R) were found to be irrelevant for nimotuzumab binding, suggesting that the latter can be used to treat EGF-R-positive tumors even after resistance to cetuximab therapy has been developed^17^. The availability of agents targeting diverse epitopes is thus critical for the success of therapeutic EGF-R targeting strategies, and preserving the unique epitope specificity of each antibody is a key issue when engineering other properties.

Here we report *in vitro* directed evolution of the phage-displayed Fab fragment of nimotuzumab, resulting in the generation of two new antibodies that keep the original epitope specificity of the parental one and show moderate affinity increases to EGF-R (3 and 3.6-fold respectively). Such differences are translated into distinctive functional properties, as both antibodies have a greatly enhanced ability to inhibit EGF-R signaling cascade. These molecules are ideal tools to study the influence of affinity on nimotuzumab effects, and could expand the usefulness of nimotuzumab-derived antibodies to additional applications.

## Results

### Nimotuzumab-derived Fab variants having an increased EGF-R binding ability were selected from a phage-displayed library

**Figure 1:**
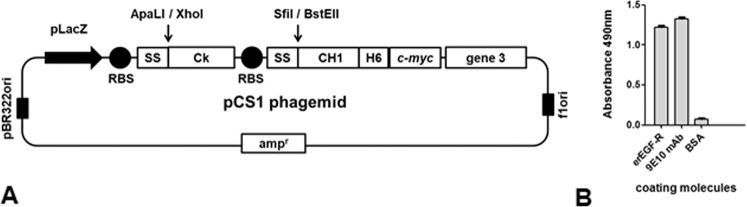
Phage display of Fab fragments derived from nimotuzumab. pCS1 phagemid vector is represented in (**A**). The vector contains pBR322 ori and f1 ori (replication origins for double and single strand DNA), an ampicillin resistance gene (amp^r^), and a bicistronic expression cassette containing the LacZ promoter, two ribosome binding sites (RBS), two signal sequence-coding genes (SS), and the genes coding for human C_K_ and CH1 human antibody constant domains. The latter was fused to sequences coding for a 6-His tag, the *c-myc* tag peptide and full-length phage PIII protein. Nimotuzumab light chain variable region gene was cloned between ApaLI and XhoI restriction sites (downstream of the M13 gen III SS), and heavy chain variable region gene was cloned between SfiI and BstEII (downstream of the pelB SS). Purified phage particles displaying nimotuzumab-derived Fab fragments were tested by ELISA (**B**) on polyvinyl chloride microtiter plates coated with the anti-*c-myc* tag 9E10 mAb, a recombinant protein comprising the extracellular region of the human EGF receptor (erEGF-R), and the unrelated protein BSA. Bound phages were detected with an anti-M13 mAb labeled with horseradish peroxidase.

Nimotuzumab variable regions had been previously displayed on filamentous phage in the form of single chain Fv (scFv) fragments^17^. Even though affinity maturation in this format was attempted before (unpublished results), the chosen platform for paratope optimization in the current work was based on phage display of Fab fragments containing nimotuzumab variable regions fused to constant domains. The rationale behind this strategy was the expectation that any modified binding site evolved in that way would have a similar architecture to a natural paratope in the whole antibody format, thus facilitating the construction of the final recombinant antibodies. Cloning of nimotuzumab variable region genes in the pCS1 phagemid vector (Fig. 1A), followed by phage rescue, resulted in successful display of Fab fragments as proven through recognition by Myc1-9E10 mAb against the *c-myc* tag (fused to the displayed heavy chain in our system) and reactivity against the recombinant EGF-R extracellular region (erEGF-R) in enzyme-linked immunosorbent assay (ELISA) (Fig. 1B). This experiment showed the suitability of Fab format for *in vitro* manipulation of nimotuzumab paratope.

**Figure 2:**
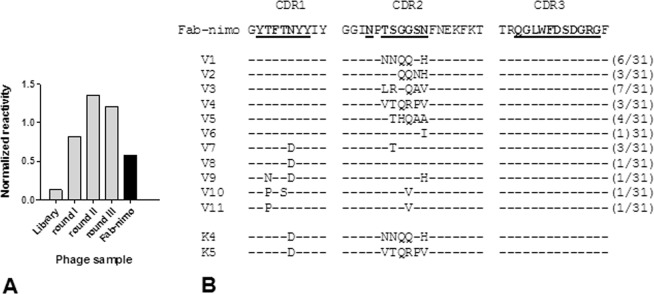
Selection of mutated variants of nimotuzumab-derived Fab fragment by panning on EGF receptor extracellular region. CDR residues of the nimotuzumab heavy chain variable region were soft-randomized in a phage-displayed Fab library. Three rounds of phage selection were performed on the immobilized extracellular region of EGF receptor (erEGF-R) recombinant protein. The reactivity of rescued phage pools from the original library and from the different selection rounds was tested by ELISA on polyvinyl chloride microtiter plates coated with the human erEGF-R recombinant protein and with the anti-*c-myc* tag 9E10 mAb. The original phage-displayed Fab fragment derived from nimotuzumab (Fab-nimo) was included as a control. Bound phages were detected with an anti-M13 mAb conjugated to horseradish peroxidase. Normalized reactivities were estimated dividing the signal obtained with the human erEGF-R by the reference signal (measured with the anti-tag mAb) and allow the comparison of target recognition abilities of the different phage pools, despite variations in protein display levels. (**A**) Colony screening after the third selection round was used to choose 31 clones with the highest normalized reactivities. Their V_H_ genes were sequenced (V1-V11), and the deduced protein CDR sequences were aligned. (**B**) The original CDR sequences of nimotuzumab are shown at the top, with those residues that were soft-randomized in the library highlighted in bold and underlined. Short lines represent conservation of the original residue at a given position. Numbers between parentheses indicate the frequencies of each mutated sequence among the set of 31 clones with the highest normalized reactivities. Two additional sequences (K4 and K5, bottom) were constructed by combining some of the selected mutations.

Replacing the original nimotuzumab V_H_ gene in the Fab construct by a synthetic collection of V_H_ variants derived from it by soft randomization of the three complementarity determining regions (CDRs)^17^ resulted in a library of 1.5 × 10^7^ variants. Most CDR positions, displaying solvent-exposed side chains, were soft-randomized, which means that the introduction of a diverse mixture of amino acids (aa) at each location was always biased towards the predominance of the residue present in the original Fab. This was accomplished by setting the probability of keeping the original nucleotide at each coding DNA position to 90% during gene library synthesis, resulting in most library members having the original residue at a given position. Each mutated V_H_ thus has a limited number of replacements, whereas every amino acid can be present at every targeted position in the library as a whole. Fab fragments with improved binding were enriched by panning on immobilized erEGF-R recombinant protein (Fig. 2A). While the initial library showed a decreased target reactivity that can be attributed to the presence of mutations affecting crucial residues involved in binding, after selection rounds on the antigen the reactivity of the phage population (normalized for display levels determined with the anti-tag 9E10 mAb) was even higher than the one of the control nimotuzumab-derived phage-displayed Fab fragment (Fab-nimo). This result indicated an increase of the intrinsic affinity in at least some selected phages. Sequencing of a sample of selected phagemids from clones with the highest normalized reactivities revealed that mutated variants arising from selection (11 unique sequences in total) can be classified in two groups: one comprising several mutations in the CDR2, and a set having replacements in both CDR1 and CDR2 (Fig. 2B).

It is remarkable that no mutations in the V_H_ CDR3 were observed among selected binders despite the presence of variants with mutations in 11 CDR3 positions in the original library (Q95, G96, L97, W98, F99, D100, S100A, D100B, G100C, R100D and G100E), which is consistent with the already observed crucial importance of this segment for target recognition by nimotuzumab^17^. Only the replacement of the codon CAG (coding for Q95) by the TAG amber stop codon (a functionally silent change in suppressor E strains like TG1 that is frequently retrieved from synthetic libraries) was repeated five times among the set of best binders. The lack of diversity in V_H_ CDR3 was a target-selected feature and not the consequence of any artifact during library construction, as sequencing of a sample of the synthetic library clones revealed the presence of codons encoding aa different from the original one at every position diversified by design (from 10 to 26% of replacements at the different CDR3 positions among library members). The number of non-original residues encoded at each position within this segment ranged between four and ten. The alternative explanation that CDR3 conservation could be related to a bias in gene expression and/or protein display, rather than to its involvement in binding, was ruled out because sequencing a sample of non-selected clones already known to display an antibody fragment (as assessed with the anti-tag 9E10 mAb) showed the expected levels of V_H_ CDR3 diversity (from 10 to 26% of aa replacements at the targeted positions, with 4–9 non-original residues appearing at each one).

On the other hand, the attempts to obtain variants with increased binding ability from total randomization of light chain nimotuzumab CDRs repeatedly failed, which is consistent with the already described minor involvement of this region in the interaction^17^.

**Figure 3:**
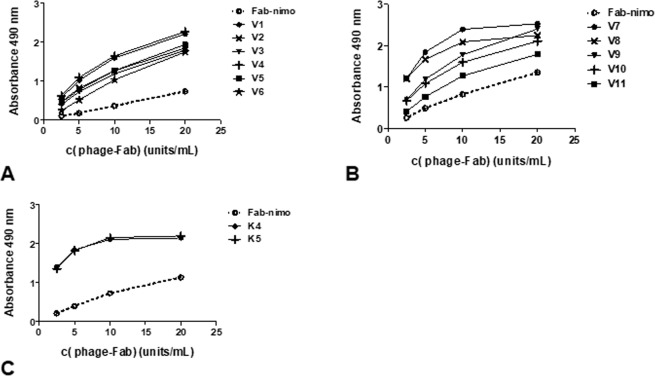
Reactivity of phage-displayed mutated variants derived from nimotuzumab Fab. Purified phage particles were tested by ELISA on polyvinyl chloride microtiter plates coated with the recombinant protein comprising the extracellular region of human EGF receptor. Bound phages were detected with an anti-M13 mAb conjugated to horseradish peroxidase. Selected variants having mutations only in CDR2 (V1-V6) are represented in (**A**), while variants with replacements in both CDR1 and CDR2 (V7-V11) are shown in (**B**). Two new variants constructed by combining some of the selected mutations are represented in (**C**). The original nimotuzumab-derived phage-displayed fragment (Fab-nimo) was included as reference (dotted line). Dilutions of phage samples were calculated to reach equivalent concentrations of all the displayed proteins (display units/mL) according to a previous ELISA on polyvinyl chloride microtiter plates coated with the anti-tag antibody 9E10 to measure display levels.

The reactivity of all selected phage-displayed mutated variants from both V_H_ groups was higher than that of wild-type Fab-nimo phage, as shown by dose-response curves in ELISA (Fig. 3A,B). For these experiments, phage samples were appropriately diluted to reach equivalent concentrations of all the displayed proteins (display units/mL), allowing a fair comparison of their respective binding abilities. This previous phage normalization procedure was based on quantitation of every displayed protein in a sandwich ELISA using the anti-*myc* tag 9E10 mAb for coating and an anti-M13 antibody conjugated to horseradish peroxidase for detection. Different displayed proteins were thus measured independently of their intrinsic target-binding properties^22^.

Two additional variants (K4 and K5) were constructed by Kunkel mutagenesis^23^, combining the CDR2 sequences of the most reactive clones from the first group (V1 and V4) with the most frequent CDR1 mutation (N31D), and shown to have increased binding ability to erEGF-R (Fig. 3C), showing the compatibility of these replacements to work together. K4 and K5 variants were chosen for further characterization.

It is noteworthy that, despite certain degree of variability in the protein display levels between different phage preparations, the display levels of selected clones were in the same range that the one of the original phage-displayed Fab-nimo. None of them showed an improved display. This finding implies that the primary driving force behind selection was target binding, and no other advantageous features like increased gene expression, improved protein periplasmic secretion and assembly on phage particles, and higher thermodynamic stability of the displayed protein. This result is in contrast with other examples of *in vitro* evolution where target-unrelated properties have been concurrently selected^24^.

### Whole recombinant antibodies including *in vitro* evolved V_H_s showed a higher affinity than nimotuzumab

Cloning K4 and K5 V_H_ genes into a mammalian cell expression vector containing the human IgG1constant domains’ gene, followed by co-transfection of NS0 myeloma cells with this construct and a vector that contains the original nimotuzumab *kappa* light chain gene, gave rise to cell lines producing the new mAbs K4 and K5. The average yields of the antibodies were 18 mg/L for nimotuzumab, 9 mg/L for K4 and 23 mg/L for K5. A comparison by size-exclusion chromatography did not reveal any improvement in the aggregation status after *in vitro* evolution. While both nimotuzumab and K5 were predominantly monomeric (94.4 and 93.2% on average, respectively), K4 showed a major peak corresponding to 80.7% of the monomeric protein and almost 20% of large aggregates. There were no manufacturability advantages for the new antibodies as compared with the original one, as it has been observed for other molecules arising from directed evolution, which can have improved stability and solubility, and subsequently increased secretion levels^24^. In fact K4 seemed to be less produced and more aggregation-prone than nimotuzumab. Developability attributes of K5 did not differ from those of the parental antibody. These observations reinforced the idea that the selective advantage of the new variants during *in vitro* evolution was related to target affinity.

The new antibodies were directly compared with nimotuzumab in terms of intrinsic affinity, after papain digestion of the three molecules to obtain monovalent Fab fragments (suppl. Figure 2), ideally suited for BIAcore measurements. Table 1 shows modest but consistent increases in binding affinity to immobilized erEGF-R protein for K4 and K5 (3- and 3.6-fold respectively). Both k_on_ and k_off_ were moderately improved upon *in vitro* affinity maturation. Suppl. Figure 3 illustrates the obtained sensorgrams. These results indicate that the affinity evolution already achieved in the phage-displayed Fab version was recapitulated in the final format of whole recombinant IgG produced in a mammalian cell host.

**Table 1:** Binding affinity and kinetic parameters of recombinant antibodies.

	k_on_ (L/mol s)	k_off_ (1/s)	K_D_ (mol/L)	Increase
nimotuzumab	1.9 ± 0.2 × 10^4^	1.3 ± 0.1 × 10^−3^	6.74 ± 1.1 × 10^−8^	^—^
K4 mAb	2.8 ± 0.5 × 10^4^	6.1 ± 0.9 × 10^−4^	2.24 ± 0.7 × 10^−8^	3×
K5 mAb	3.3 ± 0.3 × 10^4^	6.1 ± 0.5 × 10^−4^	1.86 ± 0.28 × 10^−8^	3.6×

Fab fragments from each antibody were obtained through papain digestion. These proteins were used for kinetic measurements by surface plasmon resonance on immobilized EGF receptor extracellular region recombinant protein. The values represent the mean ± SD calculated from four independent BIAcore experiments.

### *In silico* exploration revealed clues of the molecular bases of the affinity improvements

**Figure 4:**
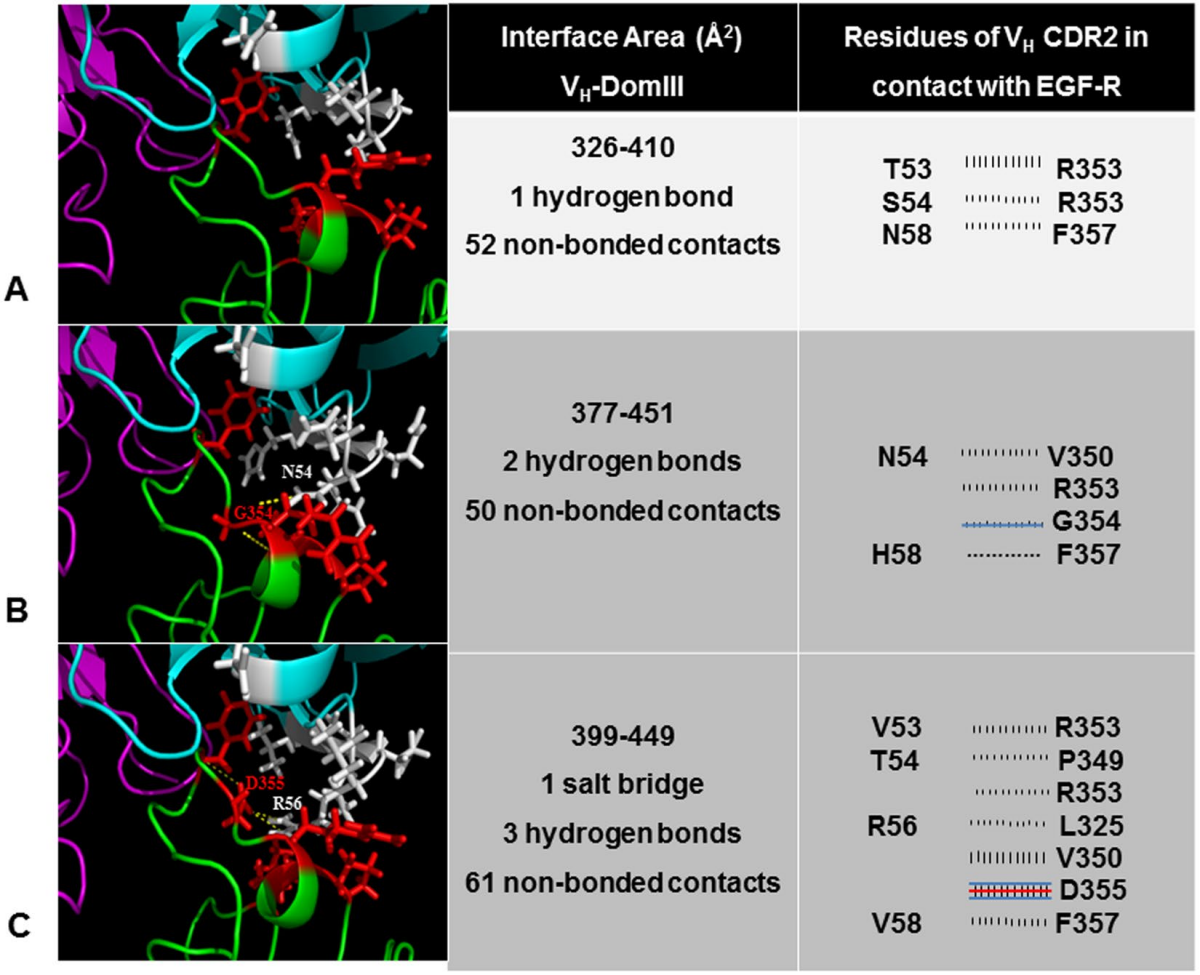
*In silico* analysis of the *in vitro* evolved interaction interfaces between antibodies and EGF receptor. Cartoon representations of nimotuzumab (**A**), K4 mAb (**B**) and K5 mAb (**C**) in complex with the antigen are shown in the left panel. The model structure of the original complex between nimotuzumab and EGF-R had been obtained from docking studies.^17^Models of the complexes between the antigen and the mutated antibodies (K4/K5) were generated by introducing the required amino acid replacements in the previous model and refining the structures. The images are centered in the interface region that involves V_H_ CDR2 residues (the segment that accumulated mutations as a result of *in vitro* affinity maturation). Heavy and light chains, and EGF-R, are colored cyan, magenta and green, respectively. Side chains of V_H_ CDR2 residues that were mutated in the current study (positions 53–58) are represented with white sticks. Side chains of EGF-R in contact with V_H_ CDR2 (<4Ǻ) are represented as red sticks. Hydrogen bonds and salt bridges are shown with yellow dotted lines. The pictures were generated with Pymol. The central panel shows the calculated contact areas between V_H_ (left number) and EGF-R domain III (right number), as well as the number of predicted interactions between them, according to the analysis with the program PDBsum implemented in an online server (http://www.ebi.ac.uk/pdbsum). Details of such interactions (only those involving V_H_ CDR2 residues targeted by mutagenesis) are represented in the right panel. Each line means an interaction between one residue in the antibody (left column) and another amino acid in the antigen (right column). Blue and red lines represent hydrogen bonds and salt bridges, respectively. Striped lines correspond to non-bonded interactions, the width of the striped line is proportional to the number of atomic contacts.

The starting point to reproduce *in silico* the interaction between the antibodies and the EGF-R was the previously reported model of nimotuzumab-EGF-R domain III binding, constructed on the bases of mutagenesis scanning data of both antigen and paratope to identify crucial residues^17^. Introduction of the new residues found in the affinity-matured versions of V_H_ and subsequent refinement of the structures led to models of the interactions between domain III and K4/K5. Both K4 and K5 are predicted to have a larger contact area with the antigen than nimotuzumab according to these modeling studies (Fig. 4). While the original residues at positions targeted by mutagenesis in nimotuzumab V_H_ CDR2 have short side chains and only T53, S54 and N58 are able to contact the epitope in the model, amino acids arising in mutated variants have in general longer side chains potentially able to establish additional interactions with the target antigen.

According to modeling studies, K4 mAb incorporates three new interactions with the antigen, including non-bonded contacts and one hydrogen bond between N54 in the V_H_ CDR2 and G354 in the EGF-R (Fig. 4B). K5 mAb, the highest affinity variant obtained in the current study, established seven new interactions with the antigen, including non-bonded contacts, and one salt bridge and two hydrogen bonds between R56 in the V_H_ CDR2 and D355 in the antigen (Fig. 4C). The new network of predicted interactions between mutated V_H_ CDR2 in affinity-matured variants and the target could explain the observed affinity increases. The selected replacement in V_H_ CDR1 is predicted to be far from the contact area, and its functional contribution remains to be elucidated. Remarkably, besides the new contacts established by mutated V_H_ CDR2, K4 kept 10/10 interactions involving critical residues within V_H_ CDR1 and CDR3 of nimotuzumab, and K5 conserved 9/10 such interactions. The above result provided a first indication that the binding mode was not essentially changed upon affinity maturation.

One of the major drawbacks of this *in silico* approach is that the new models were not constructed on the basis of an experimentally determined antigen-antibody structure, but on a previous model of the nimotuzumab-EGF-R complex^17^. Despite the accuracy of the used backrub protocol to predict side chain positions and local conformational changes upon the introduction of mutations, reliability of our results depends mainly on the quality of such an initial structure. The fact that the model is consistent with the experimental results of mutagenesis exploration of both epitope and paratope^17^, and the absence of an experimentally determined complex structure, made it a reasonable starting point for the current study. In order to establish a fair comparison between the original model and the new models obtained after *in silico* mutagenesis of the paratope, the backrub protocol was not only applied to the new binding sites, but also to the original one, allowing re-accommodation of the side chains.

### Epitope mapping studies of K4 and K5 mAbs on phage-displayed EGF-R domain III reproduced the results obtained for nimotuzumab

Even though the above described *in silico* study provided the first clues to the recognition of similar epitopes by nimotuzumab and its affinity-matured variants, experimental epitope mapping could produce strongest evidence of epitope conservation. As data from mutagenesis scanning of phage-displayed domain III surface had previously allowed delineating a detailed functional picture of the interaction between nimotuzumab and its target^17^, epitope mapping of the new K4/K5 mAbs was also attempted in the same experimental setting. Recognition of the collection of mutated phage-displayed variants with changes targeting the crucial residues (R353, S356, F357, T358 and H359) that contribute to nimotuzumab epitope formation, as well as substitutions affecting neighbor positions (L325, T330, K333) was tested by ELISA. The tolerance profile to mutations in the target antigen obtained for K4 and K5 mAbs resembled the one obtained with the parental antibody (Table 2). Residues S356 and H359 of EGF-R were absolutely required for binding. Multiple replacements (including conservative substitutions) at these positions abolished recognition by both new variants as well as by nimotuzumab. The functional contribution of aromatic residues at position 357 and hydrophobic moieties at position 353, and the minor contribution of T358 were also conserved along the antibody series. Some differences in the effect of mutations at positions 353, 357 and 358 were found (mainly between K5 mAb and the other two antibodies). K5 (and less frequently K4) tend to be more tolerant to some mutations than the original antibody. This can be explained by the fact that in the context of a stronger interaction, the individual contribution of each side chain to binding is expected to be lower. The similarities between the global tolerance profile to mutations in the antigen of nimotuzumab and their affinity-matured variants indicate that *in vitro* evolution did not result in a drastic modification of the location and chemical nature of the targeted epitope, which is the essential requirement of a directed evolution process aimed at modulating affinity without disturbing fine epitope specificity.

**Table 2:** Epitope mapping using mutagenesis scanning of phage-displayed EGF-R domain III.

	R3 (parental mouse antibody of nimotuzumab)			K4 mAb			K5 mAb		
aa in EGF-R	NT	PT	Tol	NT	PT	Tol	NT	PT	Tol
L325	—	—	A, K, P, R, S, V	—	—	A, K, P, R, S, V	—	—	A, K, P, R, S, V
T330	C	—	D, L, Q, S, W	C	—	D, L, Q, S, W	—	C	D, L, Q, S, W
K333	C	V	I, L, M, Q, R, S	C	—	I, L, M, Q, R, S, V	—	—	C, I, L, M, Q, R, S, V
R353	H, P, Q, S, T	—	K, L, M, W	H, P, Q, T	S	K, L, M, W	P	H, S, T	K, L, M, Q, W
S356	I, L, P, Q, R, T	—	—	I, L, P, Q, R, T	—	—	I, L, P, Q, R, T	—	—
F357	C, K, L, M, Q, R, S, T, V	—	Y	C, K, L, M, Q, R, S, T, V	—	Y	C, K, R, S, T, V	—	L, M, Q, Y
T358	F, G, P	E, H, Q, R, W	S	P	E, F, G, Q, R, W	H, S	P	E, F, G, Q, R, W	H, S
H359	L, P, Q, R, S, T, Y	—	—	L, P, Q, R, S, T, Y	—	—	L, P, Q, R, S, T, Y	—	—

Phage-displayed single-mutated variants of domain III were evaluated by ELISA on microtiter plates coated with the different anti-EGF-R mAbs and with the anti-*c-myc* tag 9E10 mAb. Normalized reactivities were obtained by dividing the signal obtained with a given antibody by the reference signal (measured with the anti-tag mAb). Relative reactivities (%) were calculated using the ratio between normalized reactivity of each mutated variant and the normalized reactivity of the non-mutated phage-displayed domain III. Those replacements leading to a relative reactivity below 50% were considered to be non-tolerated (NT), while mutations producing a relative reactivity in the range 50–75% were classified as partially tolerated (PT). Tolerated substitutions (Tol) were those present in variants keeping more than 75% relative reactivity. Rows corresponding to amino acids belonging to the functional nimotuzumab epitope are shaded, with the two crucial residues S356 and H359 highlighted in dark grey.

### Further mutagenesis studies on mammalian cell-produced EGF-R confirmed that K4 and K5 mAbs keep nimotuzumab fine specificity

**Figure 5:**
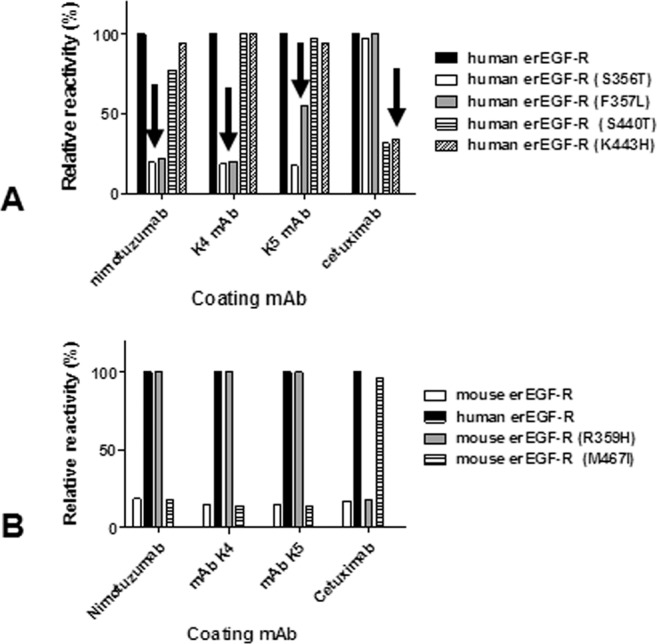
Epitope mapping through mutagenesis scanning of recombinant EGF receptor produced in HEK293-6E human cells. The reactivities of His-tagged mutated variants of human (**A**) and mouse (**B**) extracellular region of EGF receptor (erEGF-R) were evaluated on microtiter plates coated with nimotuzumab, cetuximab, K4 mAb and K5 mAb. Bound proteins were detected with an anti-His tag mAb followed by an anti-mouse Fc antibody conjugated to horseradish peroxidase. Relative reactivity (%) of each mutated protein was calculated taking the reactivity of the human non-mutated antigen as the reference. Arrows indicate loss of recognition of certain mutated variants.

Mutagenesis-based epitope mapping studies on the whole extracellular region of EGF-R produced as a recombinant soluble protein in human host cells would have the additional value of synthesizing the antigens in their natural context and incorporating native post-translational modifications like glycosylation. This refined experimental setting was used to confirm the effects of a selected set of mutations already known to abolish binding of nimotuzumab, their affinity-matured variants, and cetuximab as an additional well-characterized control antibody. The results were consistent with those obtained using phage-displayed EGF-R domain III (Fig. 5A). The conservative replacement S356T abrogated recognition by nimotuzumab, and also abolished recognition by K4/K5, reinforcing the critical contribution of Ser at this position. On the other hand, F357L abolished reactivity of both nimotuzumab and K4, but produced a partial loss of binding of the highest affinity K5 antibody. As expected, none of these variants showed a decreased recognition by cetuximab, which recognizes another functional epitope on EGF-R domain III. This result validated the specificity of mutation effects on a particular epitope. In a similar fashion, the conservative replacements S440T and K443H (targeting crucial positions for cetuximab binding) abolished recognition by this antibody, but not by nimotuzumab nor by its affinity-matured variants.

Conclusions from loss-of-recognition mutagenesis experiments can be strongly supported by additional gain-of-recognition approaches^25^, because positive results are less prone to ambiguous interpretations derived from global effects of some mutations on protein structure and folding. Therefore, the epitopes recognized by nimotuzumab and the closely related K4/K5 variants, as well as by cetuximab, were rebuilt on the scaffold of a previously non-recognized protein (mouse EGF-R). This was accomplished by introducing the residues of the human receptor that had been previously shown to determine the lack of cross-reactivity of nimotuzumab and cetuximab with its mouse counterpart, into the latter. Humanization of nimotuzumab epitope (through the single conservative mutation R359H) on mouse EGF-R thus resulted in recognition of this antigen by nimotuzumab and not by the control antibody cetuximab, whereas humanization of the cetuximab epitope (M467I) led to specific recognition only by this antibody. Remarkably, the behavior of K4/K5 in such epitope recapitulation experiments was exactly the same exhibited by nimotuzumab (Fig. 5B), providing further confirmation that the identity of the recognized epitope did not change along the affinity maturation process.

### Affinity increases translated into enhanced inhibitory capacity of K4 and K5 mAbs on EGF-R signaling

**Figure 6:**
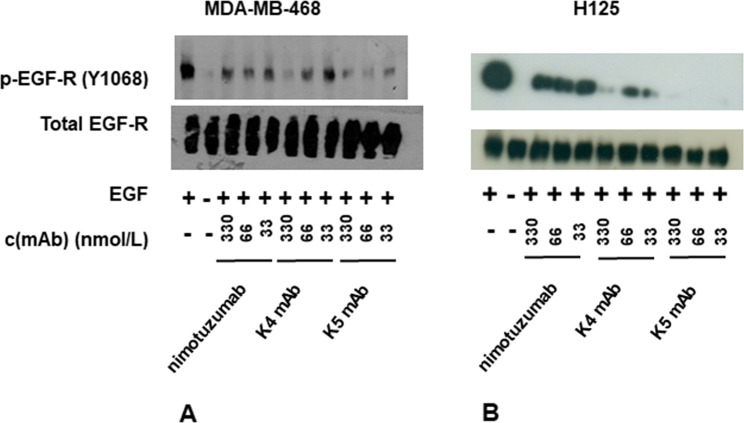
Inhibition of EGF receptor phosphorylation by antibodies. MDA-MB-468 (**A**) and H125 cells (**B**) were deprived of serum overnight and then incubated 2 h in the absence or presence of several concentrations of mAbs. Cells were stimulated with human EGF (100 ng/ml) for 10 minutes. Non-stimulated cells were used as negative control. Cell lysates were analyzed by western blot with antibodies to the Tyr 1068-phosphorylated form of EGF receptor (pEGF-R). Total EGF-R was also detected with specific antibodies.

The capacity of the affinity-matured K4/K5 mAbs to inhibit EGF-mediated phosphorylation of the cytoplasmic receptor tail (the first step in the signaling cascade initiated by stimulation of the EGF/EGF-R axis) was enhanced in comparison to the one of nimotuzumab (Fig. 6). This was shown by western blot experiments with an anti-phospho-EGF-R using lysates from cells treated with EGF in the presence or absence of neutralizing antibodies. Increased phosphorylation inhibition ability was evident for both new antibodies in experiments with cells displaying high EGF-R levels (MDA-MB-468 cell line, Fig. 6A), as well as intermediate levels (H125, Fig. 6B). The effects were associated to direct inhibition of EGF-R activation rather than to receptor internalization and degradation, as the total receptor levels (assessed with a control anti-EGF-R that does not depend on target phosphorylation status for recognition) did not vary upon antibody treatments. Future studies of receptor internalization induced by the antibodies could be performed using a suitable experimental setting, with longer treatment of the cells (8 h or more) as previously described^26^.

Remarkably, the moderate affinity increases of K4/K5 mAbs (3 and 3.6-fold in comparison with nimotuzumab) were translated into clear differences in the biological outcome of signaling inhibition assays, particularly in H125 cells. Even the difference between the two affinity-matured variants (which only reaches 1.2-fold), resulted in a distinguishable biological activity, being the highest affinity variant (K5 mAb) able to reduce receptor phosphorylation in H125 cells by more than 80% at every tested concentration in the range 33–330 nmol/L. Phosphorylation started to increase with K5 mAb concentrations of 17 nmol/L and lower (data not shown).

## Discussion

Affinity is a key property of antibodies that definitely influences their biological activity and effectiveness as therapeutic and/or diagnostic agents. In the case of anti-cancer antibodies, increasing binding affinity could in principle result in higher tumor antigen detection sensitivity, extended presence at the tumor site, requirement of lower doses to obtain therapeutic effects and increased drug efficacy. Nevertheless, the relationship between antibody affinity and clinical success is much more complex, depending on a fine balance between accumulation at the tumor site, distribution within the tumor itself as well as on healthy tissues, anti-tumor effects and undesired toxicity.

The affinity increases obtained in the current study (3–4-fold) were moderate in comparison to other reported examples also based on phage display, in which dramatic affinity improvements of two and even three orders of magnitude were achieved^27–30^. However, there were two major reasons to continue working with such affinity-matured variants instead of looking for additional affinity increases with a more stringent mutagenesis/selection strategy. First, these modest affinity improvements have a clear effect on antibody function, as shown by the stronger capacity of these antibodies to inhibit ligand-mediated receptor phosphorylation. The differences were more pronounced in the case of H125 cells (having moderate levels of EGF-R expression), which are less susceptible to receptor blockade by the original antibody nimotuzumab, probably due to the requirement of a higher antigen density for effective binding of this intermediate affinity antibody^31^. The new antibodies (K4 and K5) are thus functionally distinguishable from the parental mAb and have the potential to exhibit higher anti-tumor effects and/or extend these effects to a broader range of target cells. Translation of rather small affinity differences into significant advantages for practical applications have indeed been observed in other cases^32^.

A second reason to explore the usefulness of the new antibodies without further binding optimization is that extremely high affinity values are not necessarily equivalent to better clinical results. In fact, the best toxicity profile among anti-EGF-R mAbs currently used in the clinical setting is exhibited by nimotuzumab- the lowest affinity antibody in this group and the only one that can be chronically administered to combat cancer^3^. Such a favorable outcome of nimotuzumab therapy has been attributed to its intermediate affinity, that could result in an adequate balance between anti-tumor potency and pharmacodynamics^33^. Mathematical modeling predicts that there is an optimal affinity window for anti-EGF-R mAbs (with K_D_ values between 10^−8^ and 10^−9^ mol/L)^8^. According to this model, antibodies within this range are efficiently localized to the tumors, while their uptake by normal tissues with lower EGF-R expression is very limited. Lower affinity antibodies would not bind to the tumor, and antibodies with higher affinities would have an increased uptake by normal tissues throughout the body, thus reducing the therapeutic index and increasing toxicity. This is in agreement with the already demonstrated exquisite sensitivity of nimotuzumab to antigen density on the cell surface^14^. Remarkably, the new mAbs developed during the current work exhibit affinity values higher than the one of nimotuzumab, but still in the theoretically ideal range and below other therapeutic antibodies like cetuximab and panitumumab. Therefore, exploring their potential usefulness to target EGF-R-positive tumors deserves further attention. The search for antibodies with an intermediate affinity between nimotuzumab and cetuximab has recently led to the emergence of Ame55, an antibody with lower toxicity and better efficacy than cetuximab^34^, but differences in the epitope specificity of these antibodies preclude the establishment of a direct link between affinity and functional properties. On the other hand, K4 and K5 mAbs reported here keep nimotuzumab fine specificity and are thus suitable tools to study the contribution of affinity to both anti-tumor and toxic effects.

Similar antibody series with varying affinities against a given epitope have been successfully used to study the relationship between affinity and tumor targeting in the case of HER-2 (a structurally similar receptor belonging to the same family as EGF-R)^35,36^. Tumor retention was not significantly increased with improvements in antibody affinity beyond 10^−9^ mol/L. While the lowest affinity variant exhibited diffuse tumor staining, the highest affinity one was retained in perivascular areas of the tumor. This is consistent with the idea of the existence of an antigen barrier that prevents tumor penetration of mAbs and diminishes their therapeutic efficacy, particularly for those high affinity antibodies that bind quickly to the most accessible peripheral tumor tissue and are internalized without reaching the inner part of the tumor^37^. The effects of such heterogeneous distribution in the tumor provides additional support to the requirement of a careful affinity optimization (not always affinity increases) to improve targeting *in vivo*, and illustrate the potential advantages of intermediate affinity variants like K4 and K5.

The choice of an affinity maturation strategy is related to both the magnitude of affinity increases to be obtained and the probability of epitope conservation. A more aggressive diversification of the original variable regions’ sequence could in principle lead to larger binding improvements, but also implies a high risk of selecting totally new binding sites that recognize epitopes distinct from the original one on the same target antigen. Total and even partial CDR randomization are in fact methods for the construction of synthetic libraries, from which antibodies to virtually any epitope can be selected^38^. As the goal of the current work was to increase the affinity within a narrow range while keeping the unique fine epitope specificity already described for nimotuzumab, a careful diversification strategy was designed to explore a restricted sequence space around the original one, including new binding sites close to nimotuzumab paratope. Rational design relying upon the knowledge of the crystal structure of the complex *or in silico* optimization based on computational modeling of the complex structure has been used to manipulate other antibodies with precision mutagenesis^39,40^. In the case of nimotuzumab, crystallization of the complex has not been yet possible, but a computational model of the interaction, based on detailed mutagenesis scanning of epitope and paratope surfaces, is available. According to such previous research, the major functionally contributing amino acids from the antibody side are located in the V_H_ region (particularly in the CDR3), while the V_L_ only plays a minor role^17^. We decided to apply a combinatorial strategy that took into account *in silico* structural exploration, but also gave the paratope the opportunity to evolve *in vitro* towards solutions not predicted *a priori*. V_H_ CDRs were soft-randomized, while V_L_ CDRs were totally randomized in four separate libraries. V_L_ libraries did not produce variants with increased binding ability, reinforcing the idea that this domain is not directly engaged in the interaction. Selection from the V_H_ CDRs-diversified library resulted in absolute conservation of the V_H_ CDR3 residues (already known to be critical for binding). The strict conservation of V_H_ CDR3 strongly suggested that the *in vitro* evolved variants retained the original topology of paratope:epitope interaction, a feature considered crucial for biological function. The use of soft-randomization to screen a large number of mutations into variable domains, without disturbing intensively the binding mode by the presence of multiple mutations in every single molecule, has been reported in other examples of fine modulation of antibody binding properties^41^. Some mutations in the V_H_ CDR1 were found, while most changes were seen in the V_H_ CDR2. Significantly, based on the available models, those mutations could be predicted to create additional interactions that ultimately increase the strength of the antigen-antibody reaction. Another crucial element during affinity maturation processes, besides the degree of diversification, is the format of the molecule to be displayed. Phage display and directed evolution have been successful with both and scFv^42,43^ and Fab^28,44,45^ antibody fragments. Even though the nimotuzumab-derived scFv had been previously constructed and shown to be functional^17^, early attempts to perform affinity maturation in this format failed due to difficulties in recapitulating the increases in binding ability of the phage-displayed scFvs in whole antibodies constructed with the selected variable regions (unpublished results). Despite the many successful examples of affinity maturation in the scFv format and subsequent conversion to whole antibodies^46,47^, some authors have found an influence of the format used for affinity maturation of antibody fragments on their subsequent conversion to whole IgG molecules^48^, an effect observed for nimotuzumab as well.

As previously explained, one of the first pre-requisites established at the beginning at this work for a successful affinity maturation was the conservation of the nimotuzumab fine epitope specificity. Some authors have developed competition-based affinity maturation strategies to guarantee epitope conservation^49^. In the case of anti-EGF-R antibodies, besides being the only way to segregate the study of the functional consequences of affinity differences from the influence of recognition of different epitopes on the same target, keeping the uniqueness of nimotuzumab epitope is relevant in light of the growing number of mutations that are emerging upon anti-EGF-R treatments and mediate epitope loss and tumor resistance to therapy^17–21,50^. Targeting a clinically relevant epitope that is different to those recognized by cetuximab and panitumumab represents a valid alternative to continue the treatment of such patients. Affinity maturation strategy was oriented in that way through low-degree diversification of functionally relevant segments, as described above. Finally, the new variants were carefully analyzed to verify epitope conservation. As a first step, the epitope mapping study previously performed for nimotuzumab (based on the screening of phage-displayed mutated variants of domain III) was reproduced for K4 and K5 antibodies^17^. The results resembled the ones with the original paratope. This phage-based epitope mapping approach has been validated in several cases with excellent results^17,25,51–53^. However, it can always be argued that an antigen fragment produced in *E.coli* and fused to the phage coat might have subtle differences from the natural antigen affecting certain epitopes. Therefore, the current work introduced a further step, and some mutations already known to disrupt nimotuzumab epitope (identified on phage) were also tested in the context of the whole EGF-R extracellular domain produced by human host cells. Reproduction of results on this antigen scanning system, much more similar to the natural antigen, reinforced the idea that the epitope recognized by K4 and K5 mAbs is closely related to the nimotuzumab epitope.

A definite proof of the identity of the epitope recognized by K4 and K5 came from gain-of-recognition experiments, also performed with mammalian cells-produced antigens. This epitope mapping approach is very powerful because it does not rely on deleterious mutations that could influence epitope topology directly or indirectly^51,52^. Instead of destroying the epitopes by mutagenesis of the target antigen, single mutations at positions known to be crucial for recognition were used to recapitulate nimotuzumab epitopes on a previously non-recognized scaffold (recombinant mouse EGF-R extracellular domain). “Humanizing” the two residues responsible for the lack of cross-reactivity of both nimotuzumab and cetuximab was enough to achieve specific reconstruction of each epitope. Recapitulation of nimotuzumab epitope also resulted in recognition by K4 and K5, showing the coincidence between the molecular topology of antigen: paratope interaction within this antibody group. Both the use of mammalian cell-produced antigen variants and the application of a gain-of-recognition approach provided further support to the previous definition of the nimotuzumab epitope itself, and represent steps forward in our epitope mapping strategy of anti-EGF-R mAbs, that could be useful for multiple antibodies.

In summary, the two new mAbs obtained in the current work (K4 and K5) keep nimotuzumab fine specificity, but show moderate affinity increases that result in an enhanced capacity to inhibit EGF-R phosphorylation upon ligand binding. The impact of this change in the *in vivo* activity against diverse cancer cells, with different EGF receptor expression levels, remains to be elucidated. *In vitro* evolved paratopes could also be useful for applications where fine-tuning of the balance between binding to tumor cells and healthy tissues is crucial, like the construction of chimeric antigen receptors (CAR)-armed T cells and bispecific T-cell engagers, and tumor imaging studies.

## Methods

### Phage display of the antigen-binding fragment of nimotuzumab (Fab-nimo)

The genes coding for the heavy and light chain variable regions of nimotuzumab were amplified by polymerase chain reaction (PCR) and cloned in the pCS1 phagemid vector (Fig. 1A) using suitable restriction sites (SfiI and BstEII for V_H,_ ApaLI and XhoI for V_L_). Fab-nimo-displaying phage particles were rescued as described^17^.

### Construction of combinatorial libraries and phage selection of nimotuzumab-Fab-derived variants

The pool of soft-randomized V_H_ genes contained in a previously synthesized scFv library (GENEART)^17^ was amplified by PCR and cloned in the pCS1 phagemid vector (Fig. 1A) already containing the original nimotuzumab V_L_ gene (see the previous section). Details of library design are shown in suppl. Figure 1a. The resulting genetic constructs were used to electroporate TG1 *E.coli* (K12_(*lac-pro*), *sup*E, *thi*, *hsd*D5/F’ *tra*D36, *pro*A^+^B^+^, *lac*I^q^, *lac*Z_M15) electrocompetent cells in order to construct the Fab phage display library.

Four independent randomized V_L_ Fab libraries were constructed, two of them to diversify segments of the CDR1, and two additional ones for diversification of either CDR2 or CDR3. Details of library design appear in suppl. Figure 1b.Mutagenic oligonucleotides used for their construction are shown in suppl. Figure 1c. Each V_L_ CDR coding segment was replaced by a stretch of NNK triplets resulting in total randomization of the targeted positions through Kunkel mutagenesis^23^ as described, using the Fab-nimo genetic construct obtained in the previous section as the initial template. TG1 cells were electroporated with the reaction products to obtain the libraries.

Before phage selection a sample of 30 antibody fragment-displaying clones from each library was sent for sequencing to verify the correctness of library construction according to library design. After this initial quality check, phage selection on immobilized erEGF-R recombinant protein and ELISA screening of phage-displayed nimotuzumab-derived Fab variants were performed as described^17^. After three panning rounds, those clones having the highest normalized reactivity values (calculated as the ratio between ELISA signals obtained with the erEGF-R and with the anti-*c-myc* tag antibody) were chosen for sequencing. XL1-Blue *E.coli* cells (*rec*A1 *end*A1 *gyr*A96 *thi*−1 *hsd*R17 *sup*E44 *rel*A1 *lac* F´ *pro*AB l*ac*IqZ_M15 Tn10 Tet^r^) were infected with the corresponding phage-containing supernatants and used to purify plasmid DNA with the QIAprep Spin Miniprep kit (QIAGEN, USA). Phagemid inserts were sequenced by MACROGEN, Korea.

### ELISA screening of the reactivity of phage-displayed nimotuzumab-derived Fab variants

The display levels of the different purified phage preparations obtained as described^17^ were quantified by ELISA on immobilized anti-*c-myc* tag 9E10 mAb^22^. The pool of phages displaying non-mutated Fab-nimo was used as a standard (assuming a concentration of 100 arbitrary display units/mL) to assess the relative display level of the other phage preparations (also in display units/mL).

Polyvinyl chloride microtiter plates were coated overnight at 4 °C with recombinant erEGF-R, at 10 μg/mL in phosphate buffered saline (PBS). Plates were blocked for 1 h at room temperature (RT) with skim powder milk at 4% (w/v) in PBS (M-PBS). Purified phages (diluted in M-PBS at equivalent concentrations according to the previous ELISA experiment) were added to the plates and incubated during 2 h at RT. Plates were washed with 0.1% (v/v) Tween 20 (PBS-T). Bound phages were detected with an anti-M13 mAb conjugated to horseradish peroxidase (HRP) (GE HEALTHCARE, USA), appropriately diluted in M-PBS. Plates were incubated 1 h at RT. After washing the plates with PBS-T, substrate solution (500 μg/ml ortho-phenylenediamine and 0.015% hydrogen peroxide in 0.1 mol/l citrate-phosphate buffer, pH 5.0) was added. The reaction was stopped 15 min later, with 2.5 mol/l sulfuric acid. Absorbances at 490 nm were determined with a microplate reader.

### Production of recombinant whole antibodies in mammalian cells

The gene encoding the original nimotuzumab V_H_ gene, as well as the V_H_ genes of two mutated variants constructed through Kunkel mutagenesis to combine several selected mutations (K4 and K5), were cloned into the mammalian cells’ expression vector pSV-gpt^54^. Nimotuzumab V_L_ gene was cloned into pSV-hyg vector^54^. NS0 myeloma cells were co-transfected by electroporation with each combination of heavy and light chain genetic constructs. Stable clones were obtained using xanthine, hypoxanthine and mycophenolic acid as selection drugs. Positive clones producing whole recombinant antibodies were screened by ELISA on polyvinyl chloride microtiter plates coated an anti-human IgG (γ-chain specific) and detected with an anti-human *kappa* light chains conjugated to HRP. Positive cells were grown and the antibodies were purified from collected supernatants by Protein A affinity chromatography.

### Affinity measurements using BIAcore

Binding experiments were carried out using the Biacore T200 instrument and the Control software 2.0.1. A standard amine coupling kit (GE HEALTHCARE, USA) was used for covalent attachment of 4 ug/mL of erEGF-R recombinant protein to a CM5 biosensor chip in 10 mmol/L sodium acetate buffer at pH 4.5. The immobilized ligand reached a final response of 783.3 RU.

Fab fragments from each antibody to be tested were obtained through papain digestion using the Pierce Fab Preparation Kit (THERMO SCIENTIFIC). HBS-EP+ at pH 7.4 (GE HEALTHCARE) was used as running buffer for multiple cycle analysis of analyte binding at different concentrations of each purified Fab (derived from nimotuzumab, K4 mAb or K5 mAb), in the range between 2.5 and 600 nmol/L. Each sample was injected during 300 s at a flow rate of 15 µL/min. The surface was regenerated with 10 mmol/L of Gly-HCl, pH 2.5. Sensorgrams were analyzed using Biacore T200 evaluation software 3.0. Kinetic data were globally fitted to the 1:1 model.

### *In silico* analysis of the new mutated paratopes

A model of the structure of nimotuzumab paratope in complex with the erEGF-R was generated by docking protocols as previously described^17^. Models of K4 and K5 paratopes in complex with the erEGF-R were generated by substituting the original CDR1 and CDR2 V_H_ residues by the amino acids contained in the mutated antibodies. In order to refine the structures and solve steric problems, the backrub protocol (implemented in rosetta version 3.5)^55–57^ was applied over residues in the interface (within 5.0 Å distance) between V_H_ and the antigen, using the pivot residues flap. A total of 100 structures were generated upon this procedure, and the best one (according to the output scores) was chosen. Intermolecular interactions were analyzed with PDBsum program^58^, implemented in the online server http://www.ebi.ac.uk/pdbsum. This program classifies the interactions into salt bridges, hydrogen bonds and non-bonded contacts. The last class corresponds to atoms that are in close proximity (≤4 Å) to atoms of the interacting partner molecule, but are not able to establish neither salt bridges nor hydrogen bonds with them. Residues that are connected through non-bonded contacts can establish weak hydrophobic and van der Waals interactions, contributing to binding.

### Epitope mapping studies on mutated antigen

The screening of the reactivity of phage-displayed EGF-R domain III mutated variants by ELISA was performed as described in previous studies^17^.

The genes encoding the original human and mouse erEGF-R were cloned into the mammalian cells expression vector pCSE2.5-His. The mutated variants were constructed by site-directed mutagenesis using the appropriate primers and following the instructions of the Quickchange II site-directed mutagenesis kit (AGILENT TECHNOLOGIES, USA). Recombinant human and mouse erEGF-R proteins and their mutated variants fused to a C-terminal 6-His tag were produced by transient transfection of HEK293-6E cells as described^59^. Proteins were purified by ion metal affinity chromatography with a Ni-Sepharose matrix.

Polyvinyl microtiter plates were coated overnight at 4 °C with nimotuzumab, K4 mAb, K5 mAb and cetuximab at 10 μg/mL in PBS. Plates were blocked 1 h at RT with M-PBS. Purified human and mouse erEGF-R and its mutated variants (diluted at 10 µg/ml in M-PBS) were added to the plates and incubated during 1 h at RT. After washing with PBS-T an anti-His tag mouse mAb IgG1 (DIANOVA, Germany) appropriately diluted in M-PBS, was added to the plates and incubated 1 h at RT. Plates were washed with PBS-T. Bound recombinant molecules were detected with an anti-mouse Fc antibody conjugated to HRP (SIGMA, USA), appropriately diluted in M-PBS. Plates were incubated during 1 h at RT. After washing the plates with PBS-T, substrate solution was added. The reaction was stopped 15 min later with 2.5 mol/l sulfuric acid. Absorbances at 490 nm were determined with a microplate reader.

### EGF-R phosphorylation assay

Cells lines with different EGF-R expression levels were used: MDA-MB-468 (human breast adenocarcinoma with pleural fluid metastasis, high EGF-R expression), and H125 (human lung adenocarcinoma, moderate EGF-R expression). The experiment was repeated twice with each cell line.

The cells were seeded in DMEM-F12 with 10% fetal bovine serum (FBS) in 6-well plates. After confluence was reached, the medium was removed and DMEM-F12 without FBS was added. Anti-EGF-R antibodies at different concentrations (330, 66 and 33 nmol/L) were added after 16 h of incubation in the absence of FBS, and incubated during two additional hours. The medium was removed and fresh medium containing 100 ng/mL of human EGF was added. After 10 min the cells were washed with cold PBS. Cell lysates were prepared in RIPA buffer (150 mmol/L sodium chloride, 1% (v/v) Nonidet P-40, 0.5% (w/v) sodium deoxycholate and 0.1% (w/v) SDS in 50 mol/L Tris-HCl, pH 8.8) supplemented with 50 mmol/L sodium fluoride, 1 mmol/L sodium vanadate, 5 mmol/L EDTA and 1 mmol/L phenylmethylsulphonylfluoride just before use. Protein concentrations in the lysates were determined with the bicinchoninic acid protein assay kit (PIERCE, USA). Equal amounts of total proteins were applied to a 7.5% SDS-PAGE gel and transferred onto a PVDF membrane (SIGMA, USA). Anti-Phospho EGF-R (Y1068) (CELL SIGNALING, USA), and anti-EGF-R (sc-03) (SANTA CRUZ BIOTECHNOLOGY, USA), both obtained in rabbits, were used as primary antibodies. After the incubation with an anti-rabbit HRP-conjugated antibody, the signal was developed using a chemiluminescent substrate (SANTA CRUZ BIOTECHNOLOGY, USA) according to the manufacturer’s instructions.

## Supplementary information

Supplementary Information.
