# Supplementary material for: Affinity-matured variants derived from nimotuzumab keep the original fine specificity and exhibit superior biological activity
Source: Sci Rep. 2020 Jan 27;10:1194. doi: 10.1038/s41598-019-57279-w (PMC6985160; doi:10.1038/s41598-019-57279-w)
Supplement: Supplementary file 1 — Supplementary Information. [file 41598_2019_57279_MOESM1_ESM.pdf]

**Supplemental information**

**Title:**

# Affinity-matured variants derived from nimotuzumab keep the original fine specificity and exhibit superior biological activity


**Authors:**

Yaima Tundidor[1], Luis F. Ponce[1], Lisset Chao[1], Joaquín Solozábal[1], Michael Hust[2], Stefan Dübel[2], Gertrudis Rojas[1*]

**Author affiliations:**

[1] Center of Molecular Immunology, calle 216 esq 15, PO Box 16040, Atabey, Playa, CP 11300, La Habana, Cuba

[2] Technische Universität Braunschweig, Institute of Biochemistry, Biotechnology and Bioinformatics, Department of Biotechnology, Spielmannstraße 7, 38106 Braunschweig, Germany

*Correspondence should be addressed to G.R.: grojas@cim.sld.cu

**a** Diversification of nimotuzumab $V_H$ sequence in the soft randomization phage-displayed Fab library

original:QVQLQQSGAEVKKPGSSVKVSCKASGYTFTNYYIYWVRQAPGQGLEWIGGINPTSGGSNFNEKFKTRVTITADE

library: QVQLQQSGAEVKKPGSSVKVSCKASG***YTFTNYY***IYWVRQAPGQGLEWIGGI***N***P***TSGGSN***FNEKFKTRVTITADE

original: SSTTAYMELSSLRSEDTAFYFCTRQGLWFDSDGRGFDFWGQGTTVTVSS

library: SSTTAYMELSSLRSEDTAFYFCTR***QGLWFDSDGRG***FDFWGQGTTVTVSS

**b** Diversification of nimotuzumab $V_L$ sequence in four phage-displayed Fab libraries

original: DIQMTQSPSSLSASVGDRVTITCRSSQNIVHSNGNTYLDWYQQTPGKAPKLLIYKVSNRFSGVPSRFSGSGSG

library 1:DIQMTQSPSSLSASVGDRVTITC**X**S**XXX**IVHSNGNTYLDWYQQTPGKAPKLLIYKVSNRFSGVPSRFSGSGSG

library 2:DIQMTQSPSSLSASVGDRVTITCRSSQNI**XXXXXXX**YLDWYQQTPGKAPKLLIYKVSNRFSGVPSRFSGSGSG

library 3:DIQMTQSPSSLSASVGDRVTITCRSSQNIVHSNGNTYLDWYQQTPGKAPKLLIY**X**V**XXXX**SGVPSRFSGSGSG

library 4:DIQMTQSPSSLSASVGDRVTITCRSSQNIVHSNGNTYLDWYQQTPGKAPKLLIYKVSNRFSGVPSRFSGSGSG

original: TDFTFTISSLQPEDIATYYCFQYSHVPWTFGQGTKLEIK

library 1:TDFTFTISSLQPEDIATYYCFQYSHVPWTFGQGTKLEIK

library 2:TDFTFTISSLQPEDIATYYCFQYSHVPWTFGQGTKLEIK

library 3:TDFTFTISSLQPEDIATYYCFQYSHVPWTFGQGTKLEIK

library 4:TDFTFTISSLQPEDIATYYCFQYS**XXX**W**X**FGQGTKLEIK

**c** Mutagenic oligonucleotides used for the construction of $V_L$ libraries

library 1: ATTACTATGTACAATMNNMNNMNNAGAMNNACAGGTGATGGTCAC

library 2: GTACCAGTCTAAATAMNNMNNMNNMNNMNNMNNMNNAATGTTCTGACTAGA

library 3: GCTTGGCACACCAGAMNNMNNMNNMNNAACMNNGTAGATCAGCAGCTT

library 4: GGTCCCTTGGCCGAAMNNCCAMNNMNNMNNTGAATATTGAAAGCA

**Supplementary Figure 1.** Design of phage-displayed Fab libraries derived from nimotuzumab. The alignment between the original nimotuzumab variable regions and diversified molecules in the libraries is shown. Targeted positions (CDR residues with solvent-exposed side chains) are underlined in the original sequences. Diversified positions in each library are represented in bold face and highlighted in yellow. The design of the soft randomization library of nimotuzumab $V_H$ synthesized by Geneart is shown in (a), while (b) shows the design of four total randomization libraries of $V_L$ CDRs constructed by Kunkel mutagenesis. Italic letters in (a) represent mixtures of the 20 aa biased towards the predominance of the original residue at each position (soft randomization), while X represents the presence of the random mixture of the 20 aa encoded by an NNK codon at a given position in a library (b). Antisense mutagenic oligonucleotides used for the construction of $V_L$ libraries are shown in (c).

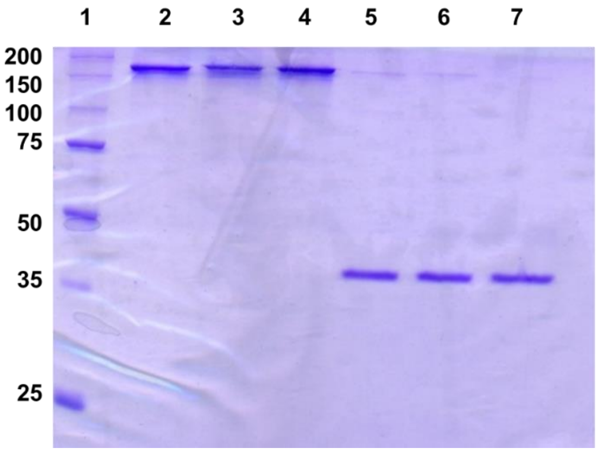


**Supplementary Figure 2.** The integrity of monoclonal antibodies and their Fab fragments was analyzed by non-reducing SDS-PAGE on a 9% gel. The molecular weight marker is shown in lane 1, with bands (kDa) identified in the left side. Protein A-purified nimotuzumab (2), K4 mAb (3) and K5 mAb (4) were analyzed. Fab fragments were obtained from the three antibodies with the Pierce™ Fab Preparation Kit (Thermo Scientific) and also analyzed. Fab fragments derived from nimotuzumab are shown in (5), and Fab fragments of K4 and K5 mAbs appear in lanes (6) and (7) respectively. The total protein amount loaded at each lane was 1 µg.

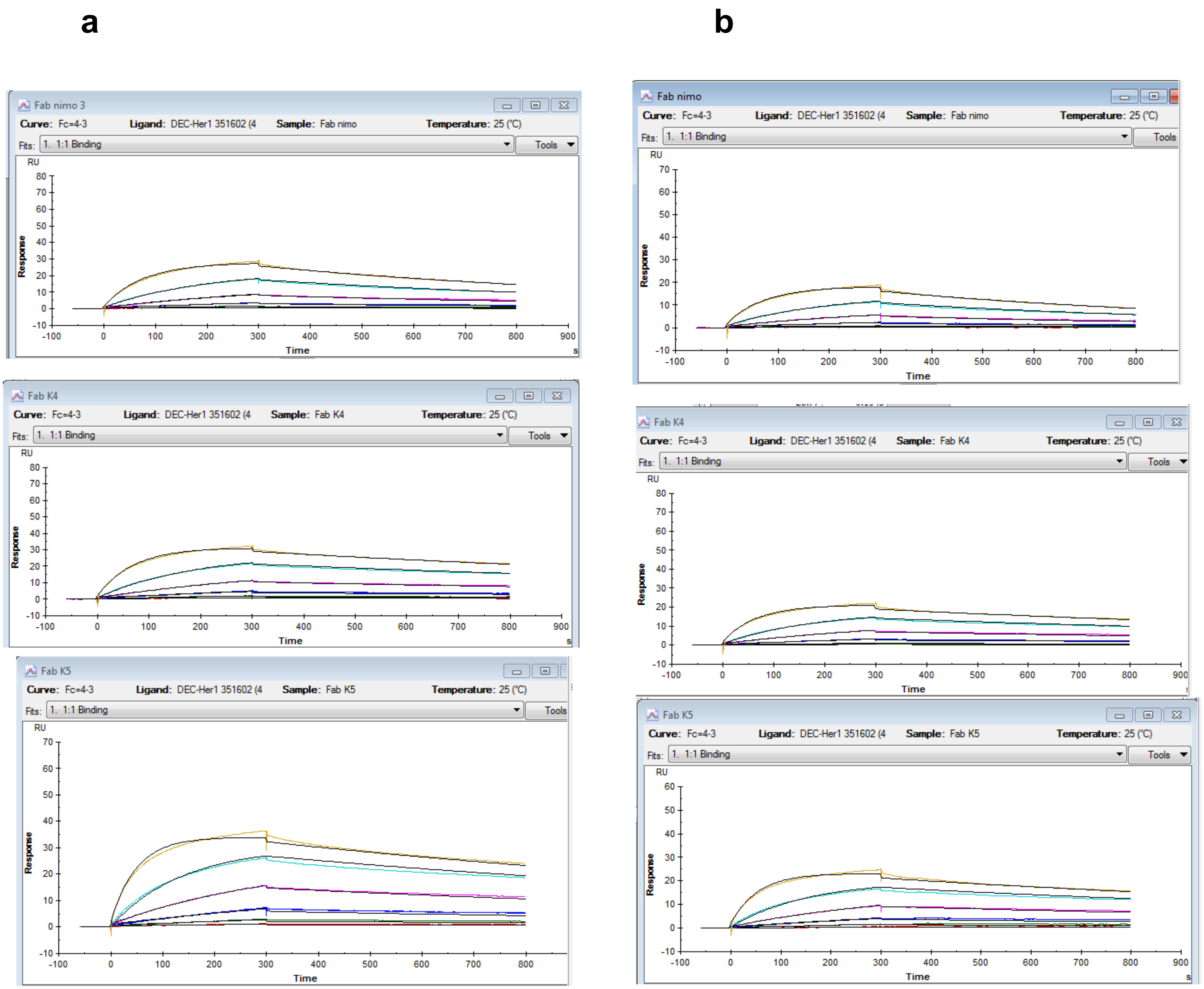


**Supplementary Figure 3.** Raw sensorgrams and fitted curves of two different experiments of affinity measurement (a and b) are shown. Binding experiments were carried out using the Biacore T200 instrument and the Control software 2.0.1. Fab preparations derived from nimotuzumab (top panels), K4 mAb (middle panels) and K5 mAb (bottom panels) were injected over a sensorchip coated with EGF-R extracellular domain recombinant protein. Sensorgrams were analyzed using Biacore T200 evaluation software 3.0. Kinetic data were globally fitted to the 1:1 model.
